# On the phylogenetic position of Myzostomida: can 77 genes get it wrong?

**DOI:** 10.1186/1471-2148-9-150

**Published:** 2009-07-01

**Authors:** Christoph Bleidorn, Lars Podsiadlowski, Min Zhong, Igor Eeckhaut, Stefanie Hartmann, Kenneth M Halanych, Ralph Tiedemann

**Affiliations:** 1 Unit of Evolutionary Biology/Systematic Zoology, Institute of Biochemistry and Biology, University of Potsdam, Karl-Liebknecht-Strasse 24-25, Haus 26, D-14476 Potsdam-Golm, Germany; 2 Institute of Evolutionary Biology and Ecology, Rheinische Friedrich-Wilhelms-Universität Bonn, An der Immenburg 1, D-53121 Bonn, Germany; 3 Department of Biological Sciences, Auburn University, 101 Life Science Building, AL 36849, USA; 4 Marine Biology Laboratory, Natural Sciences Building, University of Mons-Hainaut, Av. Champs de Mars 6, B-7000 Mons, Belgium; 5 Unit of Bioinformatics, Institute of Biochemistry and Biology, University of Potsdam, Karl-Liebknecht-Strasse 24-25, Haus 26, D-14476 Potsdam-Golm, Germany

## Abstract

**Background:**

Phylogenomic analyses recently became popular to address questions about deep metazoan phylogeny. Ribosomal proteins (RP) dominate many of these analyses or are, in some cases, the only genes included. Despite initial hopes, phylogenomic analyses including tens to hundreds of genes still fail to robustly place many bilaterian taxa.

**Results:**

Using the phylogenetic position of myzostomids as an example, we show that phylogenies derived from RP genes and mitochondrial genes produce incongruent results. Whereas the former support a position within a clade of platyzoan taxa, mitochondrial data recovers an annelid affinity, which is strongly supported by the gene order data and is congruent with morphology. Using hypothesis testing, our RP data significantly rejects the annelids affinity, whereas a platyzoan relationship is significantly rejected by the mitochondrial data.

**Conclusion:**

We conclude (i) that reliance of a set of markers belonging to a single class of macromolecular complexes might bias the analysis, and (ii) that concatenation of all available data might introduce conflicting signal into phylogenetic analyses. We therefore strongly recommend testing for data incongruence in phylogenomic analyses. Furthermore, judging all available data, we consider the annelid affinity hypothesis more plausible than a possible platyzoan affinity for myzostomids, and suspect long branch attraction is influencing the RP data. However, this hypothesis needs further confirmation by future analyses.

## Background

Molecular phylogenies based on a single or a few genes often lead to apparently conflicting signals. Violation of orthology assumption, biases leading to non-phylogenetic signal, and stochastic error related to gene length might be problematic [1]. Use of phylogenomics (molecular phylogenetic studies using a genome-scale approach) has been thought to overcome these problems, and "ending incongruence" was in sight [2]. However, poor taxon sampling [3] and systematic error that is positively misleading [4] can cause phylogenomic analyses to yield incorrect trees with high support.

Use of phylogenomic analyses to address deep metazoan relationships has recently increased. Many of these analyses consist of concatenated sets of ribosomal proteins (RP) [5-8] or of data sets dominated by RP data [3]. RP genes are highly expressed and therefore often outnumber other genes in EST-data sets. They are assumed to be largely free of paralogy across metazoans [9,10] and as such seem to represent good candidates for phylogenetic analyses.

The phylogenetic position of myzostomids, parasitic organisms typically found on echinoderms, has been highly disputed over centuries, and possible relationships with flatworms [11] or syndermatans [12] have been suggested by single gene analyses. However, analyses of mitochondrial gene order and sequence data show strong evidence that myzostomids are part of the annelid radiation [13], a result that is congruent with morphological investigations [14]. These results are contrasted by phylogenomic analyses based on an EST-borne 150 gene dataset [15] that group myzostomids within a clade of platyzoan taxa including flatworms, rotifers, gnathostomulids, and gastrotrichs. Nevertheless, the position of Myzostomida, and some other taxa, has been regarded as unstable, and Dunn et al. [15] excluded these taxa from further analyses with these EST data. Taxa that defy robust phylogenetic placement are called "problematic taxa" [16].

Here we compare analyses of two independent datasets to elucidate the phylogenetic position of Myzostomida: RP genes and mitochondrial genomes. We show that markers belonging to a single class of macromolecular complexes might bias the analysis and discuss implications for phylogenomic analyses in general.

## Results and discussion

**Figure 1:**
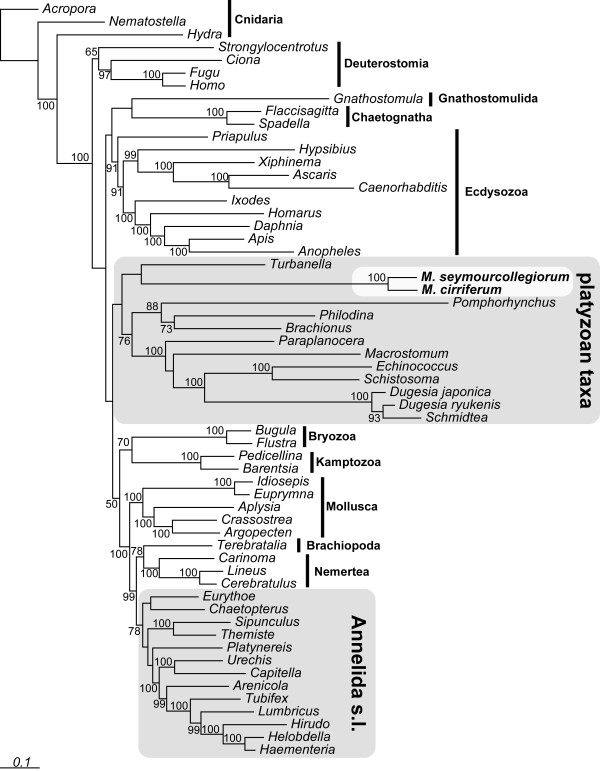
**ML analysis of the RP-dataset using RAxML with mixed models**. Bootstrap support estimated from 100 replicates is given at the nodes.

Analysing an alignment consisting of 77 RP genes, the best tree of the ML-analysis (Figure 1) supports monophyly of Myzostomida (ML-bootstrap-support (MLB) 100%). They are recovered as sister group of the gastrotrich *Turbanella *(support <50%), and together placed in a clade containing platyzoan taxa with long branches, including Syndermata (Acanthocephala + Rotifera) and Platyhelminthes (support <50%). Annelids (including echiurids and sipunculids) are recovered as monophyletic (MLB 78%). To test if this result is driven by only few genes, we performed two partition jackknifing analyses where we generated 100 concatenated datasets containing either 35 or 50 randomly drawn gene partitions. ML analyses of all these 200 newly generated datasets were conducted. We found by calculating the branch attachment frequency (BAF) for Myzostomida using Phyutilitly [17], that myzostomids group with *Turbanella *in 33% of the 35-gene datasets, and in 41% of the 50 gene dataset (see Additional File 1). Alternatively, myzostomids grouped as sister to Bilateria (24%/13%), with gnathostumulids (24%/22%), or with chaetognaths (8%/17%). Interestingly, these taxa are suspected of having high rates of nucleotide substitution. In none of these analyses did myzostomids group with annelids. These analyses also shows that the high amount of missing data (as typical for EST-based datasets), seems to have no influence regarding the phylogenetic position of the myzostomids.

These results were additionally supported by a Bayesian analysis under a site-heterogeneous model (see Additional File 1). Congruent to the ML-analysis, myzostomids grouped with *Turbanella *and cluster between long-branched platyzoan taxa. Additionally, we performed hypothesis-testing to evaluate if single gene topologies are congruent with the best ML tree of the initial concatenated 77-RP analysis. For these analyses, we pruned taxa missing in single gene datasets from the best tree and used these trees as a constraint for ML-analyses. Using AU-tests as implemented in CONSEL [18], we found that all 77 single gene analyses are congruent with the best tree. Moreover, the AU-test significantly rejects monophyly of a clade consisting of Myzostomida and Annelida sensu lato (s.l.) when analysing the complete dataset. Summarising these analyses, the RP dataset weakly supports a platyzoan/myzostomid association, without any support for an annelid origin. This relationship was also suggested by earlier molecular analyses based on a few genes [11,12].

**Figure 2:**
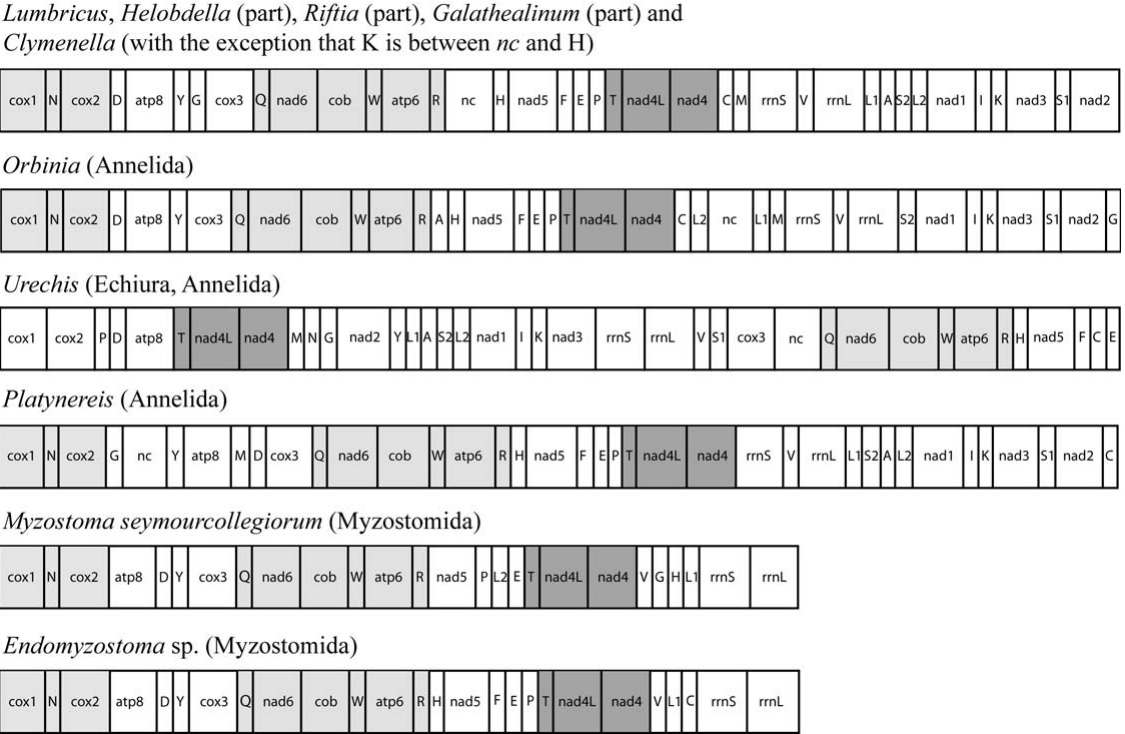
**Mitochondrial gene order of *Myzostoma seymourcollegiorum *compared with annelids**. Protein-coding genes and ribosomal RNA genes were identified by blasting on the NCBI Entrez databases. Transfer RNA genes were identified by their potential secondary structures using the tRNAscan-SE Search Server (Lowe and Eddy 1997). Identical patterns between taxa are highlighted. Abbreviations are as follow: ATP synthase subunits (*atp6*, *atp8*) cytochrome c oxidase subunits (*cox1*-*cox3*), apocytochrome b (*cob*), nicotinamide adenine dinucleotide ubiquinone oxireductase subunits (*nad1*-*nad6*), small and large ribosomal subunit (*rrnS*, *rrnL*). Transfer RNA genes are denominated by the corresponding amino acid (one letter code).

For the second data set, we sequenced another nearly complete mitochondrial genome. Within myzostomids, two major clades can be identified [19], and both are represented by the available myzostomids mitochondrial genomes (*Endomyzostoma *sp. reported here and *Myzostoma seymourcollegiorum *from Bleidorn et al. [13]). The gene order (Figure 2) of the endoparasitic *Endomyzostoma *species is similar to that of the ectocommensal *Myzostoma seymourcollegiorum *and as such reveals an order of protein coding and rRNA genes which is identical to the conserved pattern of (most) annelids, while no other animal taxon shares this pattern with myzostomids and annelids [13,20,21].

**Figure 3:**
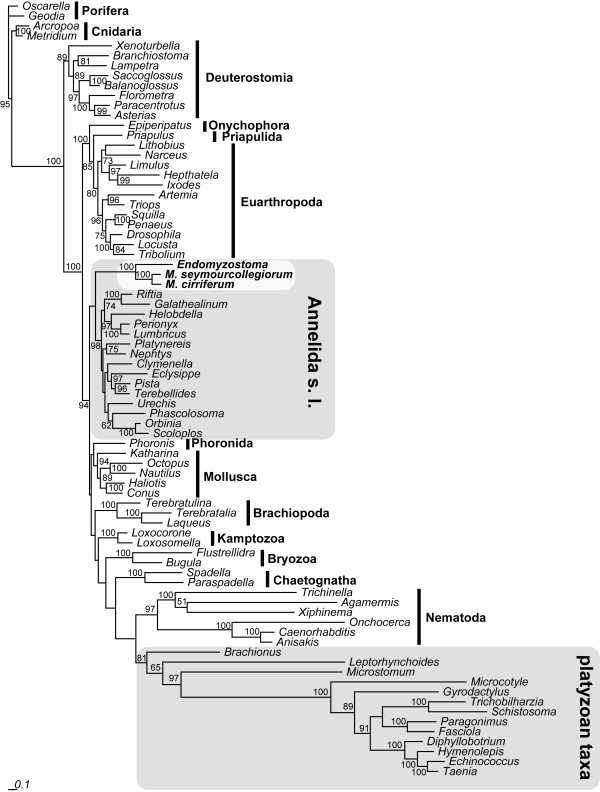
**ML analysis of the mitochondrial gene dataset**. Analysis was conducted with RAxML using mixed models. Bootstrap values from 100 replicates are given at the nodes.

ML-analysis of the 78-taxa mitochondrial genome dataset (Figure 3), including data for three myzostomids (the two mentioned above, plus mitochondrial genes found in the EST-library of *Myzostoma cirriferum*), recovers monophyletic Myzostomida (MLB 100%) as sister group to all other annelids (MLB <50%). Included platyzoan taxa (Platyhelminthes, Acanthocephala, Rotifera) form a monophyletic group (MLB 81%). Very similar results are revealed by Bayesian analysis under a site-heterogeneous model (see Additional File 1). Here, a clade containing Annelida s.l. and Myzostomida is supported by a posterior probability of 1.0.

Using hypothesis testing, we were able to significantly reject monophyly of a clade containing platyzoan taxa (Platyhelminthes and Syndermata) and Myzostomida.

The conflict regarding the phylogenetic position of myzostomids between analyses of the RP and the mitochondrial dataset is obvious – but only one of these hypotheses can be true. Consistent with the mitochondrial data, an annelid affinity is also supported by the nuclear Myosin II gene [13], *Hox *genes [22], and is in line with morphological data [14,23-25].

When accepting the results of the RP analyses, we have to assume convergent evolution of many morphological characters (e.g. chaetae, parapodia, trochophore larvae) and an exceptional case of convergence in mitochondrial gene order between annelids and myzostomids. In the other case, we have to assume that 77 RP genes are misleading phylogenetic analysis. Reasons for incongruence between markers might be either biological (e.g., selection, incomplete lineage sorting), or methodological (e.g., inaccurate phylogenetic reconstruction due to model misspecification) [26,27]. In the case of lineage sorting we would expect mixed signal when comparing the 77 RP genes. But this is not the case, as there is not any support for an annelid affinity in this dataset. Due to lack of concordance in the taxon sampling we were not able to combine both sets of markers into a single supermatrix and as such methods estimating species trees from gene trees (e.g. BEST, [28]) were not applicable. However, Ewing et al. [29] found no evidence that lineage sorting is misleading phylogenetic reconstruction by analysing a 216 gene deep metazoan phylogeny dataset.

But it might not be far fetched that analyses of RP genes are misleading. It has been shown that phylogenetic analyses of rRNA genes are affected by long-branch attraction regarding the position of myzostomids [13], and co-evolution between ribosomal proteins and its rRNA binding sites have been already demonstrated [30]. Moreover, in a phylogenomic analysis regarding Ecdysozoa, analysing different macromolecular complexes individually recover different hypotheses (e.g., RP genes supported a different hypothesis than Chaperonins) [31]. Another study on the same topic found that ribosomal proteins might be misleading due to evolutionary biases [10]. The existence of systematic functional or structural signal that competes with ancestral signal has been recently demonstrated for phylogenetic datasets [32].

Analyses by Rokas et al. [2] suggested that combining many genes in large molecular datasets will overcome problems of single gene analyses and end incongruence [33]. Despite these hopes, subsequent analysis using phylogenomic datasets [3,15] largely supported the backbone of the "New animal phylogeny" [34], but failed to resolve the phylogenetic position of many so-called problematic taxa [15,35,36]. Moreover, such analyses disagree in resolving relationships at the base of the metazoan tree [15,37].

In the case of myzostomids, our analyses show that different marker sets can resolve different topologies and usage of complete macromolecular complexes might bring conflicting signal into supermatrices and as such mislead analyses. Interestingly, we do not find any conflict within our RP dataset, but all incongruence is between both sets of markers. As such, reliance on a set of sequences belonging to a single macromolecular complex might give a biased picture, as these genes might share a common evolutionary bias. This holds true for either mitochondrial or ribosomal proteins. For future work, we strongly recommend careful inspection of phylogenomic datasets for incongruent signals [38,39] in order to refine phylogenomic analyses, as this might be the key for the placement of so-called problematic taxa.

## Conclusion

Analysing a 77 gene RP-dataset, we found that a grouping of myzostomids within platyzoan taxa is favoured. Statistical tests have shown that this is congruent with every single gene partition of this dataset and jackknifing analysis with subsequent investigation of the branch attachment frequency of myzostomids revealed no sign of support for an annelid affinity. Contrasting these results, analyses of mitochondrial sequences support an annelid affinity for myzostomids. This result is in line with some nuclear genes (Myosin II, *Hox *genes) and morphology, and is strongly supported by mitochondrial gene order and as such we consider this hypothesis more plausible than a possible platyzoan affinity.

Irrespective of which hypothesis will confirmed by future analyses, we conclude (i) that reliance of a set of markers belonging to a single class of macromolecular complexes might bias the analysis, and (ii) that concatenation of all data might introduce conflicting signal into the analyses. We therefore strongly recommend testing for data incongruence in phylogenomic analyses, as this might be the key for robust phylogenetic placement of problematic taxa.

## Methods

Individuals of *Myzostoma cirriferum *were collected from its host, the crinoid *Antedon bifida*, sampled in Morgat (France). Total RNA of ~100 frozen individuals was extracted using the Qiagen RNeasy Plant Mini Kit (Qiagen, Hilden, Germany). An amplified cDNA library was constructed at the Max Planck Institute for Molecular Genetics in Berlin using CloneMiner (Invitrogen). cDNA was size fractioned and directional cloned using the vector pDNR-LIB. Clones containing cDNA inserts were sequenced from the 5' end on the automated capillary sequencer systems ABI 3730 XL (Applied Biosystems, Darmstadt, Germany) and MegaBace 4500 (GE Healthcare, München, Germany) using BigDye chemistry. EST processing was done at the Center for Integrative Bioinformatics in Vienna. Sequencing chromatograms were evaluated using Phred [40,41]. Vector-, adapter-, poly-A-, and bacterial sequences were removed using Lucy [42], SeqClean [43], and CrossMatch [44]. Sequences were then clustered and assembled using the TIGCL package [43] by performing pairwise comparisons (MGIBlast) and a subsequent clustering using CAP3 [45]. All *M. cirriferum *EST's have been deposited in the EMBL sequence database [46].

We generated an additional nearly complete mitochondrial genome for the endoparasitic myzostomid *Endomyzostoma sp*. Individuals were collected in Antarctic peninsula region area by dredge from the *R/V Laurence M. Gould *and frozen at -80°C after collection. Total genomic DNA extractions employed the DNeasy Tissue Kit (Qiagen) according to the manufacture's instructions. The genome of *Endomyzostoma sp. *was amplified in four overlapping fragments. First, we used taxonomically inclusive primers [47] to amplify the conserved regions of *mLSU*, *cox1*, *cob *and *nad5 *genes. PCR products were purified using QIAquick PCR purification kit (Qiagen) and sequenced using a CEQ8000 (Beckmann). Three pairs of specific long-PCR primers (Table 1) were designed to amplify these long fragments: *cox1*-*cob*, *cob*-*nad5 *and *nad5-mLSU*. Long PCRs were employed on Eppendorf Mastercycler (Eppendorf) PCR machines using Takara LA-Taq PCR System. 50 μl long PCR reactions were set up including 5 μl 10×buffer, 8 μl dNTP (2 mM), 5 μl MgCl_2 _(25 mM), 2 μl of each long PCR specific primers (10 μM each), 0.5 μl Takara LA-Taq (5 U/μl), 2 μl DNA template and 25.5 μl sterilized distilled water. The long PCR protocol was 94°C for 3 min, followed by 35 cycles with 94°C for 30 sec, 53 or 54°C for 30 s, and 70°C for 12 min; final extension at 72°C for 10 min and hold at 4°C. The *cox1-cob *fragment was around 8 kb; the *cob*-*nad5 *was 2 kb, while *nad5-mLSU *was about 4.5 kb in size. These three fragments were purified using QiaQuick Gel Extraction Kit (Qiagen) and then cloned into the pGEM-T Easy vector (Promega). Positive clones were screened by PCRs and plasmids were isolated by QIAprep Spin Miniprep Kit (Qiagen). Then *EcoR*I was used to digest the isolated plasmids to check the insert size. Primer walking was employed to sequence this plasmid with large inserts.

**Table 1:** Long PCR Primers for amplifications of *Endomyzostoma *mtDNA:

**Fragments**	**Primer name**	**Sequence**	**Annealing Temp**.
*cox1-cob*	CO1-Myz-longF	5'---ATT TTT TCC TTA CAT TTA GCT GGG GCT AGG-3'	53
	Cytb-Myz-longR	5'---TGT TTA ACT CCT AAA GGG TTT GAT GAC CCG C---3'	53
*cob-nad5*	Cytb-Myz-longF	5'---TCC TCA TTA ATA AAA ATC CCG TTC CAC CCG---3'	54
	Nad5-Myz-618R	5'---TAC TAG TGC AGA AAC GGG TGT AGG TGC TGC---3'	54
*nad5-mLSU*	Nad5-Myz-615F	5'---GTA CAC TCA TCA ACA TTA GTA ACA GCA GGC---3'	54
	16S-Myz-longR	5'---CTT TAG AAA AAT AAA CCT GTT ATC CCT GTG G---3'	54

Sequences were joined together and edited using DNASTAR™ Lasergene programs SeqMan and MegAlign [48]. Blast searches were used to identify protein-coding genes and ribosomal RNA genes; tRNA genes were identified using tRNAscan-SE web server [49] under default settings and source = "mito/chloroplast", or drawn by hand based on their potential secondary structures and anticodon sequences. The GenBank accession number for the partial mitochondrial genome is FJ975144.

### Phylogenetic analyses of the ribosomal protein dataset

We used the published alignments [5,7] as backbone for our analysis. Human ribosomal protein genes retrieved from the Ribosomal Protein Gene Database [50] as search template for local tblastN searches using an e value <e-10 as threshold value for matches. We searched our EST-data of M. *cirriferum*, as well as selected EST-processed (Table 2) taxa from the NCBI trace archive [46] and the EST Database [51] for ribosomal proteins. All sequences were translated into amino acids using the program Wise2 [52].

**Table 2:** List of taxa included in the ribosomal protein dataset.

OTU	higher taxon	Genes	% AAs present
*Acropora milepora*	Cnidaria	59	60.15
*Anopheles gambiae*	Arthropoda	77	99.61
*Apis mellifera*	Arthropoda	77	99.07
*Aplysia californica*	Mollusca	76	96.46
*Arenicola marina*	Annelida	60	66.44
*Argopecten irradians*	Mollusca	70	93.71
*Ascaris suum*	Platyhelminthes	76	95.36
*Barentsia elongata*	Kamptozoa	46	54.19
*Brachionus plicatilis*	Rotifera	70	90.82
*Bugula neritina*	Bryozoa	77	98.09
*Caenorhabditis elegans*	Nematoda	77	98.99
*Capitella *sp. I	Annelida	76	86.63
*Carinoma mutabilis*	Nemertea	73	93.57
*Cerebratulus lacteus*	Nemertea	71	90.23
*Chaetopterus variegatus*	Annelida	67	84.95
*Ciona intestinalis*	Tunicata	77	99.49
*Crassostrea gigas*	Mollusca	75	94.16
*Daphnia magna*	Arthropoda	77	97.63
*Dugesia japonica*	Platyhelminthes	67	75.20
*Dugesia ryukyuensis*	Platyhelminthes	62	75.76
*Echinococcus granulatus*	Platyhelminthes	73	92.17
*Euprymna scolopes*	Mollusca	58	78.15
*Eurythoe complanata*	Annelida	41	39.93
*Flaccisagitta enflata*	Chaetognatha	61	69.58
*Flustra foliacea*	Bryozoa	76	89.93
*Gnathostomulum paradoxa*	Gnathostomulida	59	69.44
*Haementeria depressa*	Annelida	54	53.32
*Helobdella robusta*	Annelida	75	78.78
*Hirudo medicinalis*	Annelida	64	85.13
*Homarus americanus*	Arthropoda	57	70.38
*Homo sapiens*	Vertebrata	77	99.70
*Hydra magnipapillata*	Cnidaria	77	98.79
*Hypsibius dujardini*	Tardigrada	74	86.16
*Idiosepius paradoxus*	Mollusca	43	57.63
*Ixodes scapularis*	Arthropoda	71	87.07
*Lineus viridis*	Nemertea	57	73.05
*Lumbricus rubellus*	Annelida	76	98.32
*Macrostomum lignano*	Platyhelminthes	56	70.06
*Myzostoma cirriferum*	Myzostomida	47	64.84
*Myzostoma seymourcollegiorum*	Myzostomida	62	75.47
*Nematostella vectensis*	Cnidaria	72	85.36
*Paraplanoca *sp.	Platyhelminthes	70	88.46
*Pedicellina cernua*	Kamptozoa	71	89.31
*Philodina roseola*	Rotifera	28	32.29
*Platynereis dumerilli*	Annelida	26	40.54
*Pomphorhynchus laevis*	Acanthocephala	63	63.04
*Priapulus caudatus*	Priapulida	37	36.12
*Schistosoma mansoni*	Platyhelminthes	77	98.42
*Schmidtea mediterranea*	Platyhelminthes	77	97.14
*Sipunculus nudus*	Annelida	49	47.11
*Spadella cephaloptera*	Chaetognatha	66	79.94
*Strongylocentrotus purpuratus*	Echinodermata	76	94.80
*Takifugu rubripes*	Vertebrata	77	99.86
*Terebratalia transversa*	Brachiopoda	64	78.17
*Themiste lageniformes*	Annelida	64	78.06
*Tubifex tubifex*	Annelida	76	96.90
*Turbanella ambronensis*	Gastrotricha	57	57.32
*Urechis caupo*	Annelida	73	92.73
*Xiphinema index*	Nematoda	70	90.44

Number of ribosomal protein genes and percentage of amino acids present in the concatenated dataset are given.

Alignments of 77 single ribosomal genes were generated using MAFFT [53]. The software REAP [54] was subsequently used to mask all alignments prior to computing phylogenies: columns with many gaps or highly diverse amino acids were removed from the peptide alignments. A concatenated alignment of all 77 single gene alignments was constructed. The alignment has been deposited at treebase [55].

We used the AIC as implemented in ProtTest 1.3 [56] for model selection of the concatenated dataset. For Maximum Likelihood (ML) analysis, we used RAxML [57] with the PROTGAMMARTREV model to analyse single gene partitions, as well as the concatenated dataset. The concatenated dataset was analysed using mixed models for 77 single gene partitions. Clade stability was estimated by 100 replicates of non-parametric bootstrapping.

In a second step, we performed partition jackknifing analyses where we generated 100 concatenated datasets each containing either 35 or 50 randomly drawn gene partitions. ML analyses of all these 200 newly generated datasets were analysed under mixed models with the settings as described above. We calculated the Branch Attachment Frequency (BAF) for Myzostomida using Phyutilitly [17] for the 100 35-gene datasets, as well as for the 100 50-gene datasets. BAF visualizes alternative positions of particular taxa across a set of trees.

We conducted Bayesian inference based on the site-heterogeneous CAT model using PhyloBayes v2.1c [58]. Two independent chains were run were run for 17814 and 14209 points. To check for convergence, the program bpcomp [58] was used to compare the bipartitions between the two runs. With a burn-in of 1000 and taking every two trees, the largest discrepancy observed between bipartitions was 0.129. After discarding the burn-in, a majority rule consensus tree was computed using both chains to approximate posterior probabilities. We performed hypothesis testing to evaluate if single gene topologies are congruent with the best ML tree of the concatenated (77 gene) analysis. For these analyses, we pruned taxa missing in single gene datasets from the best tree and used these trees as a constraint for ML-analyses of single gene ribosomal protein datasets using RAxML, ver. 7.03 [57] with parameters described above. We computed per-site log-likelihoods with RAxML for both, the topology inferred by the single gene analysis and the constrained topology from the best tree, and used an AU-test as implemented in CONSEL [18] to test if these hypotheses differ significantly. Moreover, we constrained the monophyly of clade consisting of Annelida sensu lato (i.e. including echiurids, siboglinids, and sipunculids) and myzostomids and tested with the method mentioned above if this hypothesis differs significantly from the best tree.

### Phylogenetic analysis of mitochondrial genome sequences

Amino acid alignments of protein-coding genes from 78 complete and partial mitochondrial genomes (Table 3) were computed using ClustalW as implemented in Bioedit ver. 7.0.1 [59]. Mitochondrial sequences were downloaded from OGRe database [60]. Additionally, we performed BLAST searches to find mitochondrial genes within the newly generated EST-library of *Myzostoma cirriferum*.

**Table 3:** List of species included in the mitochondrial genome dataset. Incomplete mitochondrial genomes are indicated with an asterik (*).

OTU	higher taxon
*Acropora tenuis*	Cnidaria
*Agamermis *sp.	Nematoda
*Anisakis simplex*	Nematoda
*Artemia franciscana*	Arthropoda
*Asterias amurensis*	Echinodermata
*Balanoglossus carnosus*	Hemichordata
*Brachionus plicatilis*	Rotifera
*Branchiostoma florida*	Cephalochordata
*Bugula neritina*	Bryozoa
*Caenorhabditis elegans*	Nematoda
*Clymenella torquata*	Annelida
*Conus textile*	Mollusca
*Diphyllobotrium latum*	Platyhelminthes
*Drosophila melanogaster*	Arthropoda
*Echinococcus granolosus*	Platyhelminthes
Eclysippe vanelli *	Annelida
Endomyzostoma sp. *	Myzostomida
*Epiperipatus biolleyi*	Onychophora
*Fasciola hepatica*	Platyhelminthes
*Florometra serratissima*	Echinodermata
*Flustrellidra hispida*	Bryozoa
Galathealinum brachiosum *	Annelida
*Geodia neptuni*	Porifera
*Gyrodactylus salaris*	Platyhelminthes
*Haliotis rubra*	Mollusca
*Helobdella robusta*	Annelida
*Heptathela hangzhouensis*	Arthropoda
*Hymenolepis diminuta*	Platyhelminthes
*Ixodes hexagonus*	Arthropoda
*Katharina tunicata*	Mollusca
*Lampetra fluviatilis*	Vertebrata
*Laqueus rubellus*	Brachiopoda
*Leptorhynchoides thecatus*	Acanthocephala
*Limulus polyphemus*	Arthropoda
*Lithobius forficatus*	Arthropoda
*Locusta migratoria*	Arthropoda
*Loxocorone allax*	Kamptozoa
*Loxosomella aloxiata*	Kamptozoa
*Lumbricus terrestris*	Annelida
*Metridium senile*	Cnidaria
*Microcotyle sebastis*	Platyhelminthes
Microstomum lineare *	Platyhelminthes
Myzostoma cirriferum *	Myzostomida
Myzostoma seymourcollegiorum *	Myzostomida
*Narceus annularus*	Arthropoda
*Nautilus macromphalus*	Mollusca
*Nephtys *sp.	Annelida
*Octopus vulgaris*	Mollusca
*Onchocerca volvulus*	Nematoda
*Orbinia latreillii*	Annelida
*Oscarella carmela*	Porifera
*Paracentrotus lividus*	Echinodermata
*Paragonimus westermani*	Platyhelminthes
*Paraspadella gotoi*	Chaetognatha
*Penaeus monodon*	Arthropoda
*Perionyx excavata*	Annelida
Phascolosoma gouldii *	Annelida
Phoronis psammophila *	Phoronida
*Pista cristata*	Annelida
*Platynereis dumerilli*	Annelida
*Priapulus caudatus*	Priapulida
Riftia pachyptila *	Annelida
*Saccoglossus kowalevskii*	Hemichordata
*Schistosoma mansoni*	Platyhelminthes
Scoloplos armiger *	Annelida
*Spadella cephaloptera*	Chaetognatha
*Squilla mantis*	Arthropoda
*Taenia asiatica*	Platyhelminthes
*Terebellides stroemi*	Annelida
*Terebratalia transversa*	Brachiopoda
*Terebratulina retusa*	Brachiopoda
*Tribolium castaneum*	Arthropoda
*Trichinella spiralis*	Nematoda
*Trichobilharzia regenti*	Platyhelminthes
*Triops cancriformis*	Arthropoda
*Urechis caupo*	Annelida
*Xenoturbella bocki*	Xenoturbellida
*Xiphinema americanum*	Nematoda

Gblocks, ver. 0.91 [61] was used to identify unambiguously aligned proportions of the alignments. Parameters used were: minimum number of sequences for a conserved position = 41, minimum number of sequences for a flank position: 41, maximum number of contiguous non-conserved positions: 8, minimum length of a block: 10, allowed gap positions: with half, use similarity matrix: yes. Gblocks treatment recovered 51% of the original alignment, leading to a concatenated alignment of 2295 amino acids, with all genes except *atp8 *being partially represented in the final alignment. The alignment has been deposited at treebase [55].

Maximum likelihood analysis was performed with RaxML, ver. 7.03 [57]. MtRev + CAT was chosen as model for amino acid substitutions. The dataset was partitioned according to single gene sequences, so that model parameters and amino acid frequencies were optimized for each single gene alignment. 100 bootstrap replicates were performed to infer the support of clades from the best tree. Additionally, we constrained monophyly of a clade containing myzostomids and platyzoan taxa (Plathyhelminthes + Syndermata) and used hypothesis as described above, if this clade is significantly rejected when compared with the best tree.

We conducted Bayesian inference based on the site-heterogeneous CAT model using PhyloBayes v2.1c [58] as described above. Two independent chains were run were run for 26739 and 26660 points. With a burn-in of 15000 and taking every two trees, the largest discrepancy observed between bipartitions was 0.107.

## Authors' contributions

CB conducted labwork for subsequent generation of the Myzostoma EST-library at the MPI for Molecular Genetics in Berlin (Germany). MZ did all PCR experiments and sequencing of the mitochondrial genome and annotated the genome. CB, LP and SH performed phylogenetic analysis of sequence data. CB, KMH and RT conceived and supervised this study. CB drafted the manuscript, all other authors helped in interpretation of data and discussion of results. All authors read and approved the manuscript.

## Supplementary Material

Additional file 1 **Supplemental figures 1–3**. BAF analysis of the RP dataset, Phylobayes analysis of the RP dataset, Phylobayes analysis of the mtDNA dataset. Click here for file
