# Supplementary material for: On the phylogenetic position of Myzostomida: can 77 genes get it wrong?
Source: BMC Evol Biol. 2009 Jul 1;9:150. doi: 10.1186/1471-2148-9-150 (PMC2716322; doi:10.1186/1471-2148-9-150)
Supplement: Additional file 1 — Supplemental figures 1–3. BAF analysis of the RP dataset, Phylobayes analysis of the RP dataset, Phylobayes analysis of the mtDNA dataset. [file 1471-2148-9-150-S1.pdf]

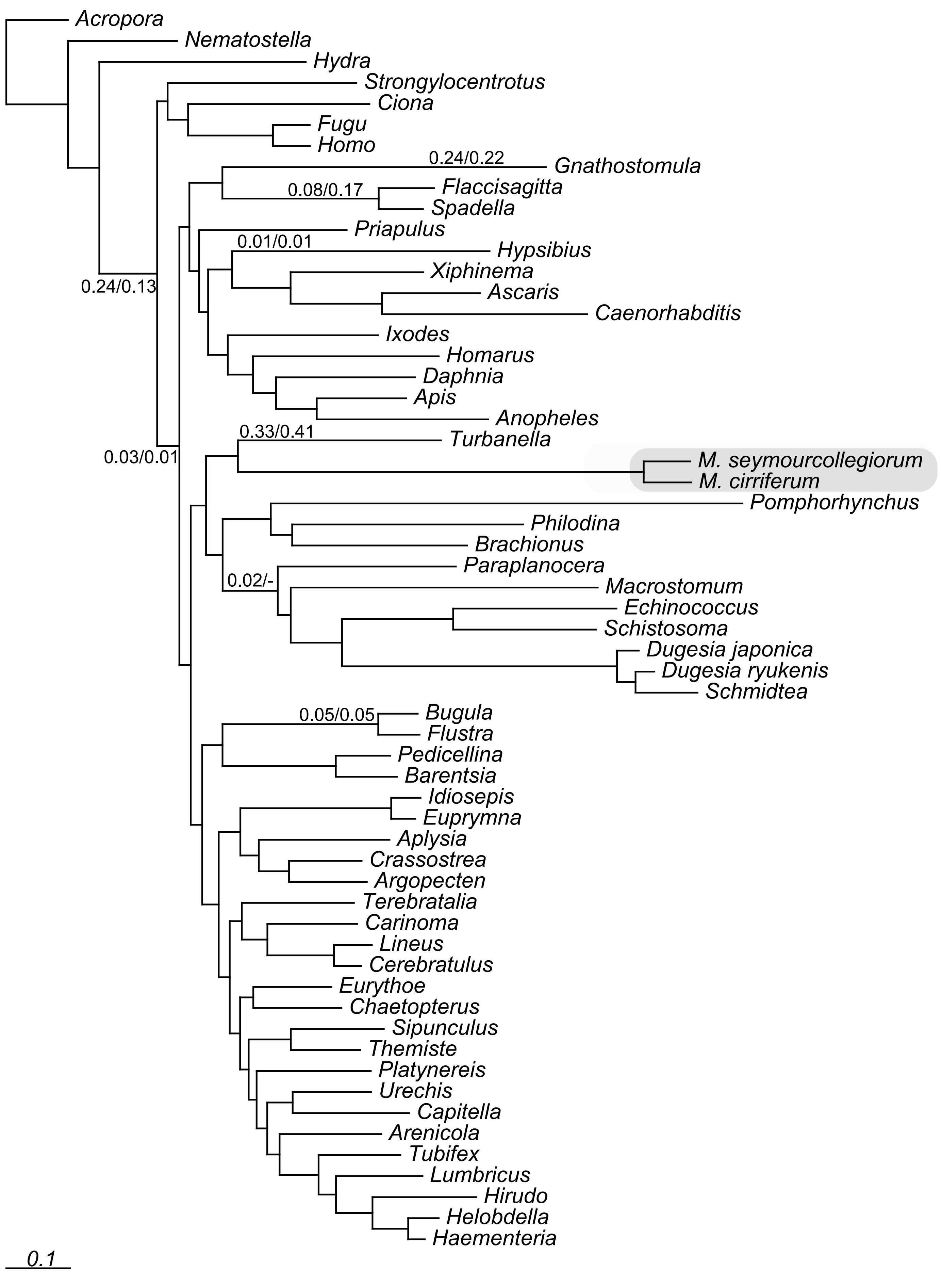

Suppl. Fig. 1: Branch attachment frequency (BAF) of the partition jackknifing analysis mapped on the best ML-tree from the concatenated RP-dataset. BAF's are given at the node, whereas the first number indicates the frequency a clade has been recovered in the 100 35-gene replicates and the second indicates the frequency fo the 50-gene-replicates.

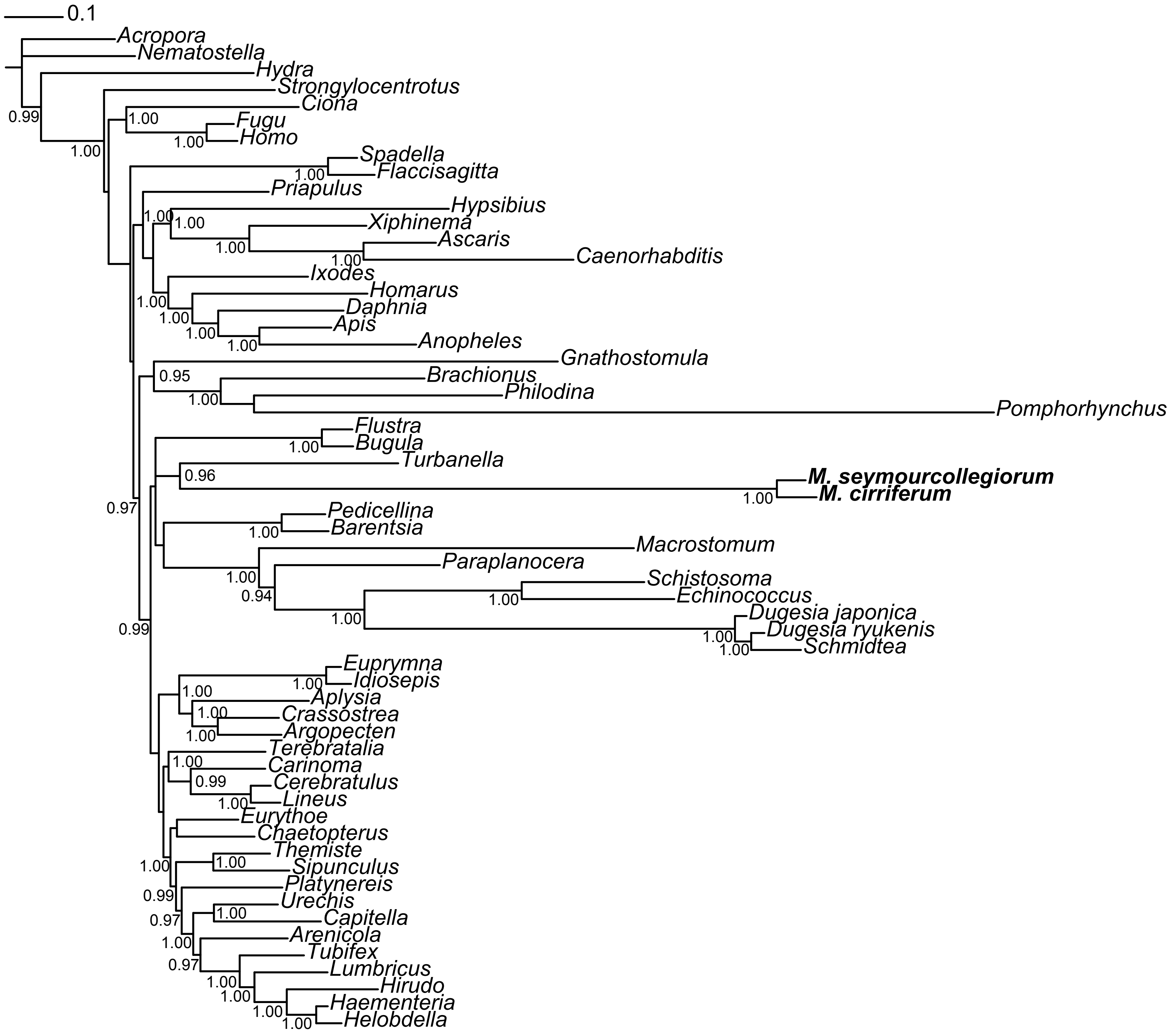

Suppl. Fig. 2: Bayesian inference of the RP-dataset based on the site-heterogeneous CAT-model using Phylobayes v2.1C. Posterior probabilities are given at the nodes.

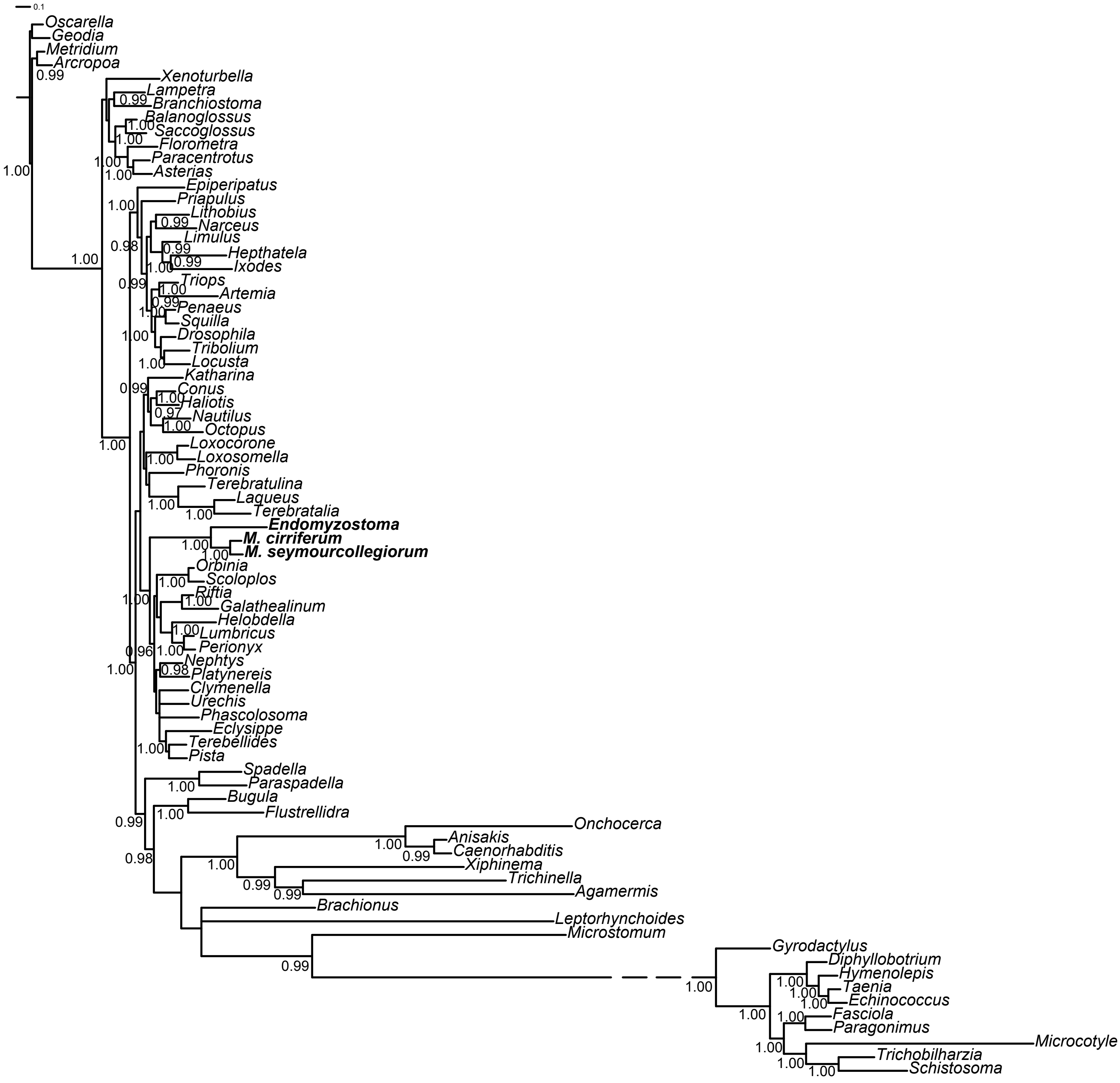

Suppl. Fig. 3: Bayesian inference of the mtDNA-dataset based on the site-heterogeneous CAT-model using Phylobayes v2.1C. Posterior probabilities are given at the nodes.
